# Zinc-alpha2-glycoprotein, dysglycaemia and insulin resistance: a systematic review and meta-analysis

**DOI:** 10.1007/s11154-020-09553-w

**Published:** 2020-05-07

**Authors:** Harriet M. Pearsey, Joseph Henson, Jack A. Sargeant, Melanie J. Davies, Kamlesh Khunti, Toru Suzuki, Kelly A. Bowden-Davies, Daniel J. Cuthbertson, Thomas E. Yates

**Affiliations:** 1 Diabetes Research Centre, Leicester General Hospital, Gwendolen Road, Leicester, LE5 4PW UK; 2 NIHR Leicester Biomedical Research Centre, Leicester, UK; 3 Department of Health Science, University of Leicester, Leicester, UK; 4 NIHR ARC East Midlands, Leicester, UK; 5 Cardiovascular Sciences Unit, Leicester Glenfeild Hospital, Leicester, UK; 6 School of Biomedical Sciences, Newcastle University, Newcastle, UK; 7 Clinical Sciences Centre, Aintree University Hospitals NHS Foundation Trust, Liverpool, UK; 8 Faculty of Health and Life Sciences, University of Liverpool, Liverpool, UK

**Keywords:** Alpha-2-glycoprotein, zinc, AZGP1, Hyperglycaemia, Dysglycaemia, Insulin resistance

## Abstract

To systematically review the current literature investigating associations between zinc-alpha2-glycoprotein (ZAG) and dysglycaemia (including type 2 diabetes (T2DM), poly-cystic-ovary syndrome (PCOS), pre-diabetes or insulin resistance). This included relationships between ZAG and continuous measures of insulin and glucose. Additionally, we performed a meta-analysis to estimate the extent that ZAG differs between individuals with or without dysglycaemia; whilst examining the potential influence of adiposity. A systematic search was performed on four databases for studies on circulating ZAG concentrations in adult human populations, comparing healthy controls to individuals with dysglycaemia. Key characteristics, including the mean ZAG concentrations (mg∙L^−1^), and any correlational statistics between ZAG and continuous measures of glucose, glycated haemoglobin (HbA1c) or insulin were extracted. Meta-analyses were performed to compare metabolically healthy controls to cases, and on studies that compared controls and cases considered overweight or obese (body mass index (BMI) ≥25 kg^.^m^2^). 1575 papers were identified and 14 studies (16 cohorts) were considered eligible for inclusion. Circulating ZAG was lower in individuals with dysglycaemia compared to metabolically healthy controls (−4.14 [−8.17, −0.11] mg^**.**^L^−1^; *I*^***2***^ = 98.5%; p < 0.001). When using data from only studies with overweight or obese groups with or without dysglycaemia (three studies (four cohorts); pooled *n* = 332), the difference in circulating ZAG was no longer significant (−0.30 [−3.67, 3.07] mg^.^ L^−1^; *I*^***2***^ = 28.0%; *p* = 0.225). These data suggest that ZAG may be implicated in dysglycaemia, although there was significant heterogeneity across different studies and the mediating effect of adiposity cannot be excluded. Therefore, more research is needed before robust conclusions can be drawn.

**Electronic supplementary material:**

The online version of this article (10.1007/s11154-020-09553-w) contains supplementary material, which is available to authorized users.

## Introduction

Type 2 diabetes mellitus (T2DM) is a chronic metabolic disease characterised by peripheral insulin resistance (IR) and inadequate relative insulin secretion, resulting in hyperglycaemia (1). T2DM encompasses approximately 90% of all cases of diabetes, with the worldwide prevalence of diabetes expected to rise from 415 million (2015) to 642 million by 2040, equivalent to more than 5% of the world’s population (2).

Excess adipose tissue, as seen in obesity, plays a central role in promoting IR and increasing the risk of developing T2DM. Adipose tissue acts as a powerful endocrine organ secreting a range of cytokines (termed adipokines) that affect whole-body physiology (3). Ectopic fat is associated with elevated levels of pro-inflammatory adipokines (e.g. tumour-necrosis factor-alpha (TNF-alpha), interleukin-6 (IL-6) and leptin) and lower levels of anti-inflammatory adipokines (e.g. adiponectin) which have been postulated to contribute to the development of IR and T2DM (4–7).

One adipokine that has come under recent scrutiny for its association with T2DM is Zinc-alpha2-glycoprotein (ZAG). ZAG is a 40–43 kDa single chain polypeptide (8) that is typically found in the majority of body fluids. Due to ZAG’s presence in a high number of body components, it has been suggested to play many roles in the body, including: fertilization, ribonuclease activity, cell adhesion, the regulation of melanin production, hindering tumour proliferation and promoting lipolysis (the hydrolysis of lipids into non-esterified fatty acids) (8). ZAG acts as an initiator of the catalytic conversion of adenosine triphosphate to cyclic adenosine monophosphate (c-AMP), which is the initial step of lipolysis (9) by binding to beta-3-adrenoreceptors on adipocyte surfaces. For this reason, ZAG has recently been categorised as a lipid-mobilising factor (10).

Although ZAG has been identified as an adipokine, its physiological effects on the risk and development of obesity-related diseases has not been fully determined. Findings have indicated that increased macrophage infiltration and the subsequent release of inflammatory cytokines, including TNF-alpha and IL-6, supresses ZAG release (11), which could be a contributor to reduced lipolysis and greater levels of ectopic fat. In line with these findings, ZAG has been shown to be positively associated with adiponectin (12, 13).

Expanding upon this evidence, elevated ZAG concentrations have been observed in cancer cachexia (14, 15), which could be a result of increased glucocorticoid levels (16). However, cancer cachexia has been associated with increased IR (17), which suggests a complex interaction of inflammatory markers.

The expanding evidence demonstrating the role of ZAG in lipid and glucose homeostasis has led to ZAG becoming a protein of increasing interest for anti-obesity and obesity-related disease therapy (18). In particular, lower levels of ZAG have been reported to be associated with the development and severity of T2DM, however the literature is contradictory, and no summary evidence has been published to date to quantify the magnitude of the association. This study aims to systematically review the current literature investigating the association between ZAG and metabolic dysfunction, identified as chronic diseases or conditions classified by hyperglycaemia or IR, referred to as dysglycaemia from here on. This includes reporting on the relationship between ZAG, insulin and glucose. Additionally, a meta-analysis will estimate the extent to which ZAG differs between individuals with or without dysglycaemia. In order to examine the potential influence of adiposity, the meta-analysis will also be restricted to only look at individuals considered overweight or obese.

## Methods

### Search strategy

This systematic review was designed according to National Institute for Health and Care Excellence (NICE) Systematic Review guidelines (19) and British Medical Journal checklist (20). The basis for this review was developed using the PICO model (presented in detail within Supplementary Materials Section 3: PICO Model Interpretation). Observational studies reporting associations between circulating ZAG concentrations (based on targeted assay analyses) and T2DM or other forms of dysglycaemia (e.g. pre-diabetes, polycystic ovary syndrome (PCOS), IR without diabetes and metabolic syndrome (MetS)), including a comparison with circulating ZAG concentrations in metabolically healthy individuals, were retrieved from four electronic databases (PubMed, Medline, Web of Science and Scopus). The inclusion of PCOS as a form of dysglycaemia in this study is due to the higher level of IR, and increased risk of pre-diabetes and T2DM when compared to the general population (21, 22). The search strategy combined keywords related to ZAG and T2DM (including common terms for dysglycaemia) (Supplementary Materials Section 3: Key words). There was no date restriction placed on the database search, but all papers must have been written in English or translated into English. Full inclusion criteria and detailed search strategy are located in Supplementary Materials (Section 3: Inclusion criteria and Selection process). Reference lists of retrieved articles were manually scanned for additional eligible studies. All aspects of literature search and study selection were performed by two researchers independently (HMP and JH).

### Data extraction

Data extraction was performed by two independent reviewers (HMP and JH). Extracted data included: descriptive information of the study (first author, date and country performed in) and participant characteristics (number of cases and controls, age and body mass index (BMI)). Data on correlational statistics for the association between ZAG and continuous measures of glucose, glycated haemoglobin (HbA1c) and insulin were also extracted. ZAG mean concentration for the groups (mg^.^ L^−1^), the standard deviation (SD) for the ZAG mean and the group sizes were taken for both the controls and cases from the least adjusted results. This allowed the raw mean difference between cases and controls to be calculated in order to conduct the meta-analysis. Further information on the data extraction process can be found in the Supplementary Materials (Section 3: Data extraction).

### Assessing risk of bias

The risk of bias assessment for studies included in this review was performed by two reviewers independently (HMP and JH) using the Strengthing the reporting of observational studies in epidemiology (STROBE) tool (23). Consensus was reached for each study with, if necessary, consultation of a third reviewer (TEY). Publication bias was assessed using funnel plots. Publication bias is important to consider as the reporting of null results can often be neglected and only results considered significant are put in to publication, which can lead to detrimental conclusions (24, 25).

### Meta-analysis

Pooled mean difference with 95% confidence intervals [95% CI] was calculated between cases and controls for circulating ZAG concentrations, using random effects models. The percentage of variability across studies attributable to heterogeneity beyond chance was quantitatively assessed using the *I*^*2*^ statistic (0% inferring no observed heterogeneity, 25% low heterogeneity, 50% medium heterogeneity and ≥ 75% infers high heterogeneity) (25). The meta-analysis was presented with data further separated into subgroups to explore pooled effects within T2DM, PCOS and “other” forms of dysglycaemia (MetS, pre-diabetes or IR without diabetes).

To mediate the effects of adiposity on the difference in ZAG between groups, a sensitivity analysis was performed by conducting a meta-analysis on the models that allowed for comparisons in overweight or obese groups. The criteria for inclusion in this restricted meta-analysis are located in the Supplementary Material (Section 3: Data extraction).

### Narrative review

A narrative review of the extracted correlational statistics (Supplementary Materials Section 2: Fig. 3) between circulating ZAG concentration and circulating markers of glucose (fasting glucose (FG), 2-h or random glucose (2 h/RG), glycated haemoglobin (HbA1c)) and insulin (fasting insulin (FIns) and homeostatic model assessment of insulin (HOMA-IR)) was performed using data from the least adjusted models. Meta-analyses were not performed due to many studies reporting incomplete data, such as presentation of *p*-values without the corresponding correlation coefficients. For ease of identification, each included study is referred to by a study number (SN). The full study titles and corresponding SN is located in the Supplementary Material (Section 1: Study numbers).

### Statistical analysis

A *p* value of <0.05 was considered statistically significant. Quantitative analysis was conducted using commercially available software STATA 15.0 (STATA, Version 15.0, StataCorp, College Station, Texas, USA).

## Results

### Systematic review

1575 studies were identified, of which 14 (16 cohorts) were included in the present review (publication dates spanning from 2008 to 2019)(13**,** 26**–**38). Further detail of the study selection process can be seen in supplementary materials (Supplementary Materials Section 2: Fig. 1). In total, 3127 participants were included across all studies, which included studies from China (Study number (SN) =1, 3, 4, 5, 10, 11, 12 and 13), Spain (SN = 6, 7 and 9), Czech Republic (SN = 2), Slovakia (SN = 8) and Egypt (SN = 14). Two studies (SN = 8 and 11) included data from more than one cohort within the same publication, whilst one study (SN = 3) allowed for data from one control group to be compared to three dysglycaemic groups (PCOS, impaired glucose tolerance and T2DM). All studies quantified ZAG using enzyme-linked immunosrobent assays (ELISA). Only two studies included in this study were prospective cohort studies (SN = 10 and 13), whilst all others were cross sectional analyses. Participants were either recruited as cases versus controls (SN = 1, 2, 3, 4, 5 and 13) or as whole cohorts which were then categorised after recruitment (SN = 6, 7, 8, 9, 10, 11, 12 and 14). Individual study sample sizes ranged from 24 (SN = 8) to 489 individuals (SN = 1). Of the studies that reported these variables, the mean (±SD) age of the individuals recruited ranged from 25.6 ± 2.2 (SN = 4) to 77.2 ± 6.1 (SN = 10) years and the mean BMI ranged from 20.50 ± 2.70 (SN = 4) to 57.32 ± 5.95 (SN = 7) kg·m^2^ across the studies. Full details of all the included studies can be found in the Supplementary Materials (Section 2: Table 1).

### Risk of bias assessment

A STROBE risk of bias assessment, performed by two reviewers independently, identified that two studies were of low quality (SN = 2 and 8), 10 were of medium quality (SN = 1, 3, 4, 5, 6, 9, 10, 12, 13 and 14) and two were of high quality (SN = 7 and 11). Common aspects were: lack of reporting on how the sample size was determined or how participants were recruited. Risk of bias for participant selection was detected in SN = 6, 7 and 8. Full details of included studies can be found in Supplementary Table 1. The funnel plots (Supplementary Material Section 2: Fig. 4 a & b), show no significant publication bias for the cohort as a whole but significant bias was identified when reporting only the overweight and obese individuals (*p* < 0.001).

### Meta-analysis

#### Absolute pooled data

When data were pooled, circulating ZAG concentrations were lower in those with dysglycaemia compared to metabolically healthy controls (−4.14 [−8.17, −0.11] mg^.^ L^−1^) (Supplementary Materials Section 2: Fig. 2a). However, findings were highly heterogeneous (*I*^*2*^ = 98.5%; *p* < 0.001). Subgroup analysis of studies categorised by the form of dysglycaemia (T2DM, PCOS and “other”) found that whilst ZAG was lower in each category compared to healthy controls there was high heterogeneity and the results for PCOS provided the only significant difference (−17.63 [−25.39, −9.87] mg^.^L^-1^).

#### Overweight and obese data only

Three studies (*n* = 332), reporting four cohorts, included comparisons between individuals with dysglycaemia and a matched overweight or obese, but metabolically healthy, control group (Supplementary Materials Section 2: Table 2 and Fig. 2B)**.** The pooled mean difference between these groups was not significant (−0.30 [−3.67, 3.07] mg^.^ L^−1^; *I*^*2*^ = 28.0%; *p* = 0.225). Full details of the cohorts included in this restricted meta-analysis can be found in the Supplementary Materials (Section 2: Table 2).

### Narrative review

Six out of 10 correlations (nine studies) reported a negative association between ZAG and FIns, whilst one reported a positive association. Circulating FG had a significant negative association with ZAG in four studies and one study reported a positive association between FG and ZAG. Only two studies reported a significant negative association with 2 h/RG measures. Four out of five studies (five out of six cohorts) that included results for HbA1c reported a significant negative association. Six studies reported significant negative correlations between ZAG and HOMA-IR, with one reporting a significant association. (Supplementary Materials Section 2: Fig. 3) (39).

## Discussion

The findings of our meta-analyses and narrative review suggest that increased ZAG is negatively associated with the development of metabolic dysfunction, particularly that related to dysglycaemia. This effect may be mediated by differences in adiposity, as there were no associations between ZAG and dysglycaemia in studies where BMI was taken into account. However, only three studies (encompassing four cohorts) were included in the latter analysis, which suggests that further research is needed in this area.

ZAG has been suggested to be an adipokine that acts as a lipid-mobilising factor playing a role in lipolysis in adipose tissue (9, 10). Reduction in the levels of ZAG could lead to lower rates of lipolysis and subsequent accumulation of lipids. This resulting lipotoxicity is recognised to result in cellular dysfunction, including impaired glucose uptake and pancreatic beta-cell dysfunction (40, 41) thus increasing the likelihood of metabolic diseases such as T2DM. The role of lipotoxicity in T2DM development is also attributed to increased inflammation through changes in cytokine profiles, which extends to adipokines (42). Therefore, if declining ZAG levels are associated with the onset of lipotoxicity, this could suggest a possible anti-inflammatory role of ZAG, which is supported by its positive association with adiponectin (13, 14, 31, 43, 44) and negative association with TNF-alpha (12, 45). Furthermore, ZAG mRNA expression in both subcutaneous and visceral adipose tissue is largely found to be predicted by adiponectin mRNA expression (33).

Along with being modulated by levels of adiposity, ZAG has also been directly implicated in the metabolic action of adipose tissue (18). For example, ZAG has been associated with reduced insulin sensitivity in visceral adipose tissue, suggesting a feedback loop whereby increased visceral adiposity reduces ZAG expression, which in turn may increase adverse metabolic functioning of visceral adipose tissue (31). A potential link been ZAG and the metabolic activity of visceral adipose tissue is important given the deleterious impact of visceral fat to metabolic health (46) and the risk of T2DM. In the opposite direction, higher levels and greater expression of ZAG have been associated with the browning of adipose tissue, which could have positive implications to metabolic health (47).

The role of ZAG in adipose tissue metabolism and the results from this meta-analyses raise the question as to whether the relationship between ZAG and dysglycaemia is in fact mediated by, or independent of, adiposity. In contrast to the findings of this meta-analysis, Yeung and colleagues investigated the difference in ZAG messenger ribonucleic acid (mRNA) and protein expression in individuals with normal glucose tolerance and T2DM patients, all of whom were overweight or obese. The mRNA expression and protein content of ZAG in adipose tissue was found to be 1.5-fold and 2.4-fold lower in those with T2DM (35), indicating that the relationship between ZAG and dysglycaemia may be independent of adiposity.

Treating rats with recombinant human ZAG has also been shown to decrease circulating levels of glucose and triglycerides, as well as increasing the expression of glucose transporter 4 (GLUT4), without a decrease in food or water intake (48). These findings are consistent with previous observations demonstrating a positive association between subcutaneous ZAG mRNA and GLUT4 mRNA expression in adipose tissue (32). However, whether ZAG's mechanistic role in GLUT4 translocation is mediated by adiposity independent pathways needs to be clarified and this may be better understood through mRNA analysis rather than circulating concentrations.

The magnitude of the influence of adiposity on other adipokines in MetS and subsequent T2DM also varies in the literature. Comparison of normal weight, overweight, obese and morbidly obese T2DM patients with metabolically healthy normal weight controls showed a difference in adipokine markers that did not exist in normal weight T2DM subjects, but the difference was exacerbated by being overweight or (morbidly) obese (49). Adiponectin, a commonly measured anti-inflammatory adipokine, has been shown to be lower in obese subjects compared to metabolically healthy normal weight controls, but there was no difference between obese subjects and T2DM patients (50). A previous systematic review and meta-analysis on circulating adiponectin and T2DM did reveal that across 15 prospective studies (14,598 participants), higher levels of adiponectin were associated with a lower risk of T2DM (51). With 13 of these studies stratified for BMI (<25 kg^.^m^-^^2^ and ≥ 25 kg^.^m^-^^2^), it was found that the relationship remained significant.

As the first study to summarise the literature surrounding the association between circulating ZAG and dysglycaemia, it identifies key limitations in the literature. Firstly, substantial heterogeneity exists, meaning more research is needed to establish whether ZAG is a valuable biomarker in the development of dysglycaemia. Secondly, there is a need to determine if the alterations in ZAG occur before the onset of dysglycaemia or vice versa. Furthermore, the summarised data shows a lack of studies isolating individuals by adiposity status to further determine if the relationship between ZAG and dysglycaemia is primarily driven by adiposity, or whether there are adiposity independent pathways linking ZAG to dysglycaemia.

This meta-analysis also has important limitations. Mainly, the inability to undertake a meta-analysis on correlation or association statistics between ZAG and markers of glucose control and IR. Additionally, there has been some investigation into the relationship between other measures of ZAG (i.e. adipose tissue mRNA and protein content) and dysglycaemia which warrants investigation.

In conclusion, this is the first study to review the current findings on circulating concentrations of ZAG and dysglycaemia. More investigations into changes in circulating ZAG with the development of dysglycaemia, with the removal of adiposity as a confounder, are needed to provide a clearer understanding.

## Electronic supplementary material

ESM 1 (PDF 990 kb)

## Data Availability

The datasets generated during and/or analysed during the current study are available from the corresponding author on reasonable request.
