# Supplementary material for: Zinc-alpha2-glycoprotein, dysglycaemia and insulin resistance: a systematic review and meta-analysis
Source: Rev Endocr Metab Disord. 2020 May 7;21(4):569–75. doi: 10.1007/s11154-020-09553-w (PMC7557496; doi:10.1007/s11154-020-09553-w)
Supplement: Supplementary file 1 — (PDF 990 kb) [file 11154_2020_9553_MOESM1_ESM.pdf]

SUPPLEMENTARY MATERIAL

Zinc-alpha2-glycoprotein, dysglycaemia and insulin resistance: A systematic review and meta-analysis

Content

1. Studies numbers (SN) assigned to each study included in the systematic review, meta-analyses and narrative review
2. Supporting Figures and Tables

   Figure 1: PRISMA flowchart

   Table 1: Table of extracted study data

   Table 2: Table of extracted study data for the overweight/ obese restricted meta-analysis

   Figure 2 (A & B): Forest plots from meta-analyses

   Figure 3: Summary of the correlational associations between continuous variables and ZAG

   Figure 4 (A & B): Funnel Plots

3. Full search methodology
   - PICO model interpretation
   - Search strategy
   - Inclusion criteria
   - Key words
   - Search term strategies
   - Numbered search strategy
   - Selection process (duplicate removal, title and abstract screening and full-paper screening)
   - Data extraction

1. Study numbers (SN)

1) Lei et al. Circulating ZAG levels are low in newly diagnosed patients with metabolic syndrome and correlate with adiponectin (2017)
2) Stejskal et al. Determination of serum ZAG in patients with metabolic syndrome by a new ELISA (2008)
3) Qu et al. The natural logarithm of ZAG/ HOMA-IR is a better predictor of insulin sensitivity than the product of triglycerides and glucose and other lipid ratios (2015)
4) Lai et al. Circulating ZAG level and insulin resistance in PCOS (2016)
5) Wang et al. Adipokine ZAG as a novel urinary biomarker presents earlier than microalbuminuria in diabetic nephropathy (2016)
6) Ceperuelo-Mallafré et al. ZAG modulates AKT-dependent insulin signaling in human adipocytes by activation of the PP2A phosphatase (2015)
7) Garrido-Sánchez et al. ZAG gene expression in adipose tissue is related in insulin resistance and lipolytic genes in morbidly obese patients (2012)
8) Balaz et al. Subcutaneous adipose tissue ZAG is associated with adipose tissue and whole-body insulin sensitivity (2014)
9) Ceperuelo-Mallafré et al. Circulating adipose tissue gene expression of ZAG in obesity: its relationship with adipokine and lipolytic gene markers in subcutaneous and visceral fat (2009)
10) Lee et al. Plasma ZAG in levels correlate positively with frailty severity in female elders (2016)
11) Yang et al. ZAG is associated with insulin resistance in humans and is regulated by hyperglycaemia, hyperinsulinemia, or liraglutide administration (2013)
12) Yeung et al. Serum zinc-alpha2-glycoprotein correlates with adiposity, triglycerides, and the key components of the metabolic syndrome in Chinese subjects (2009).
13) Zheng et al. Circulating zinc-alpha2-glycoprotein is reduced in women with polycystic ovary syndrome, but can be increased by exenatide or metformin treatment (2019).
14) Elsheik M, Elhefnawy KA, Emad G, Ismail M and Borai M. Zinc alpha 2 glycoprotein as an early biomarker of diabetic nephropathy in patients with type 2 diabetes mellitus (2019).

2. Supporting Figures

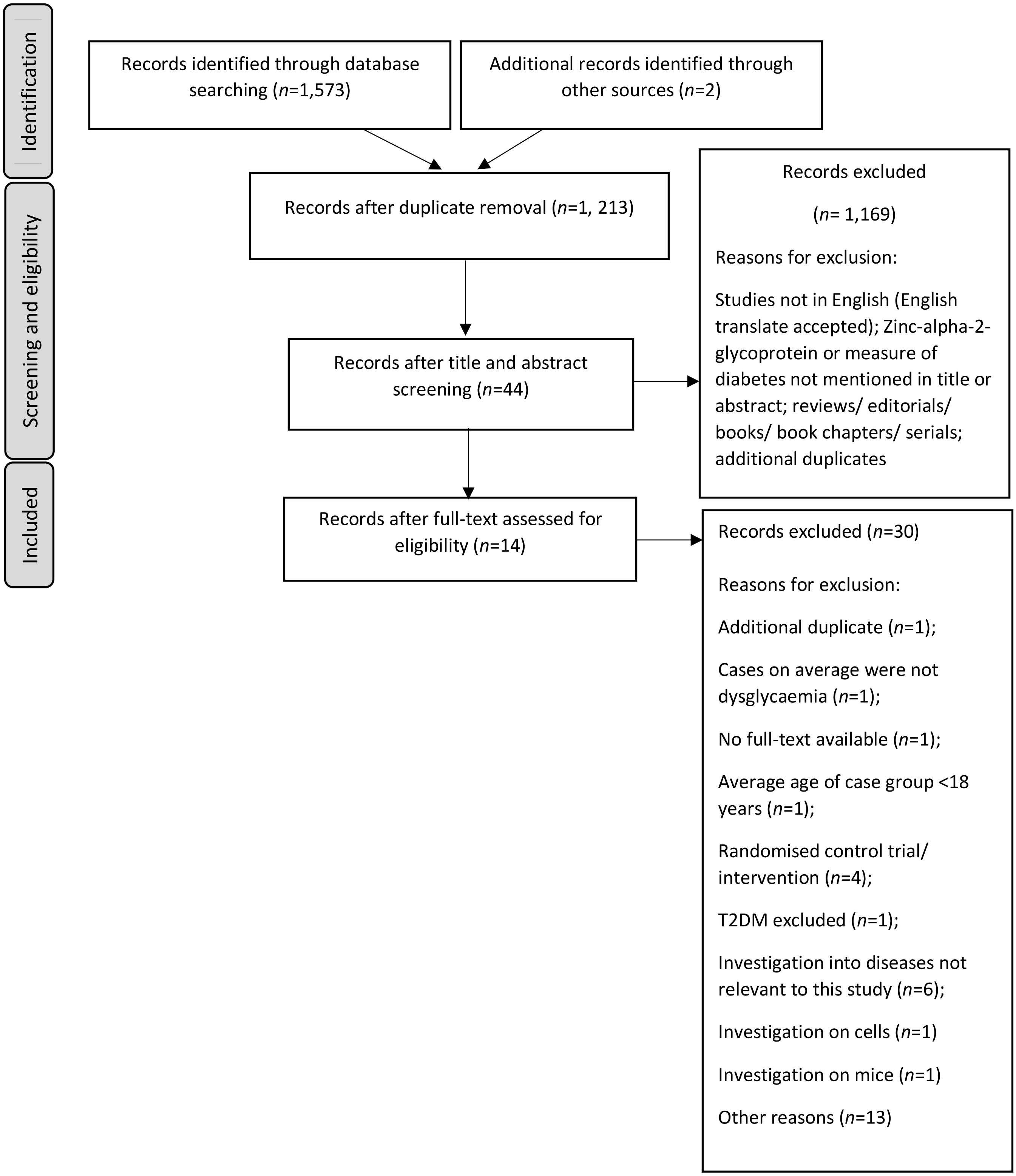

Figure 1. PRISMA flowchart depicting paper selection for inclusion in analysis.

SN	Author	Date	Country	Group	Age	BMI	ZAG mean	SD	N	Quality
1	Lei	2017	China	MetS	52.40±7.40	26.10±3.70	35.00	11.80	255	Medium
				Control	51.90±9.20	23.60±3.50	46.10	18.60	234	
2	Stejskal	2008	Czech Republic	METS	63.20±11.50	30.30±4.00	26.20	8.10	92	Low
				Non-MetS	62.80±13.00	23.90±3.00	27.40	8.30	132	
3	Qu	2016	China	Pooled control	43.00±7.00	21.59±2.90	56.43	16.10	200	Medium
				PCOS	28.00±6.00	24.37±5.36	35.31	18.28	102	
				IGT	59.00±10.00	23.05±3.30	46.72	13.40	84	
				T2DM	58.00±9.00	25.32±3.50	38.48	13.47	97	
4	Lai	2016	China	Control	25.60±2.20	20.50±2.70	53.86	15.31	100	Medium
				PCOS	26.00±4.60	24.60±4.80	35.25	18.32	99	
5	Wang	2016	China	T2DM	56.70±9.50	26.27±3.35	38.29	22.72	80	Medium
				Control	51.60±8.60	25.34±3.21	21.61	8.83	20	
6	Ceperuelo-Mallafré	2015	Spain	HOMAIR-<2	43.00±9.18	22.78±1.71	43.23	6.48	24	Medium
				HOMAIR->2-<4	50.10±14.50	23.46±2.14	42.98	9.90	9	
				HOMAIR->4	57.83±16.62	22.84±1.02	43.13	5.60	6	
7	Garrido-Sánchez	2012	Spain	High IR	38.30±7.90	57.30±5.90	37.62	9.64	14	High
				Low IR	40.90±10.60	50.60±8.10	36.21	10.33	11	
8	Balaz	2014	Slovakia	Lean/overweight (1)	36.80±1.80	23.30±0.40	53.48	9.70	21	Low
				Obese (1)	37.6±1.50	30.20±0.50	56.94	11.40	21	
				Pre-diabetic (1)	44.20±1.90	31.60±0.60	57.67	15.65	18	
				T2DM (1)	50.50±2.10	31.50±1.00	57.04	8.60	15	
				Lean (2)	37.90±1.60	21.70±1.30	48.50	12.20	6	
				Obese (2)	43.00±4.00	48.50±4.20	41.80	11.50	6	
				Pre-diabetic (2)	44.10±2.60	49.80±3.50	48.30	13.20	6	
				T2DM (2)	47.40±4.40	44.60±4.70	49.70	9.10	6	

9	Ceperuelo-Mallafré	2009	Spain	MetS	63.38±12.50	29.82±3.58	57.05	11.24	16	Medium
				Non-MetS	56.15±14.91	26.76±3.59	58.75	11.65	57	
10	Lee	2016	China	T2DM	77.19±6.12	25.05±3.38	72.28	21.90	79	Medium
				Non-T2DM			65.23	16.57	110	
11	Yang	2013	China	NGT (1)	53.00±11.00	22.66±3.15	59.36	16.20	100	High
				IGT (1)	53.00±12.00	23.79±3.27	48.84	18.74	85	
				T2DM (1)	54.00±9.00	25.31±3.48	37.14	13.25	100	
				PCOS (2)	26.40±1.90	26.30±5.51	37.84	12.47	15	
				Non-PCOS (2)	27.40±4.95	21.64±3.48	61.53	17.92	15	
12	Yeung	2009	Hong Kong	T2DM	55.10±12.5	25.40±4.10	19.20	11.50	106	Medium
				Non-T2DM			9.80	7.80	152	
				MetS			19.60	13.50	99	
				Non-MetS			10.20	8.40	159	
13	Zheng	2019	China	PCOS			51.37	1.11	182	Medium
				Non-PCOS			61.04	0.94	150	
14	Elsheikh	2019	Egypt	Control	51.00±6.62		20.27	1.52	22	Medium
				T2DM	51.00±6.91		24.55	1.68	22	
										**Pooled N= 3,127**

Table 1. Table of data extracted from the included studies included in the meta-analysis. BMI= body mass index; HOMAIR= homeostatic model assessment of insulin resistance; IGT= impaired glucose tolerance; IR= insulin resistance; MetS= Metabolic Syndrome; N= number of participants; PCOS= poly-cystic-ovary syndrome; SD= standard deviation; SN= study number; T2DM= type 2 diabetes mellitus. Where available data for age, BMI and ZAG presented as mean (SD).

SN	*Author*	*Date*	*Country*	*Group*	*BMI (kg·m²)*	*ZAG mean (mg. L⁻¹)*	*SD*	*N*
1	Lei	2017	China	Control	≥ 25.00	35.10	12.00	79
				MetS		31.40	10.10	156
7	Garrido-Sánchez	2012	Spain	Low IR	50.60±8.09	36.21	10.33	11
				High IR	57.32±5.95	37.62	9.64	14
8	Balaz	2014	Slovakia	Obese (1)	30.20±0.50	56.94	12.60	21
				Pre-diabetic (1)	31.60±0.60	57.67	15.65	18
				T2DM (1)	31.50±1.00	57.04	8.60	15
				Obese (2)	48.50±4.20	41.80	11.50	6
				Pre-diabetic (2)	49.80±3.50	48.30	13.20	6
				T2DM (2)	44.60±4.70	49.70	9.10	6
								**Pooled N= 332**

Table 2. Table of data extracted from the included studies included in the overweight/ obese only restricted meta-analysis. BMI= body mass index; IR= insulin resistance; MetS= Metabolic Syndrome; N= number of participants; PCOS= poly-cystic-ovary syndrome; SD= standard deviation; SN= study number; T2DM= type 2 diabetes mellitus. Where available data for BMI and ZAG presented as mean (SD).

A)

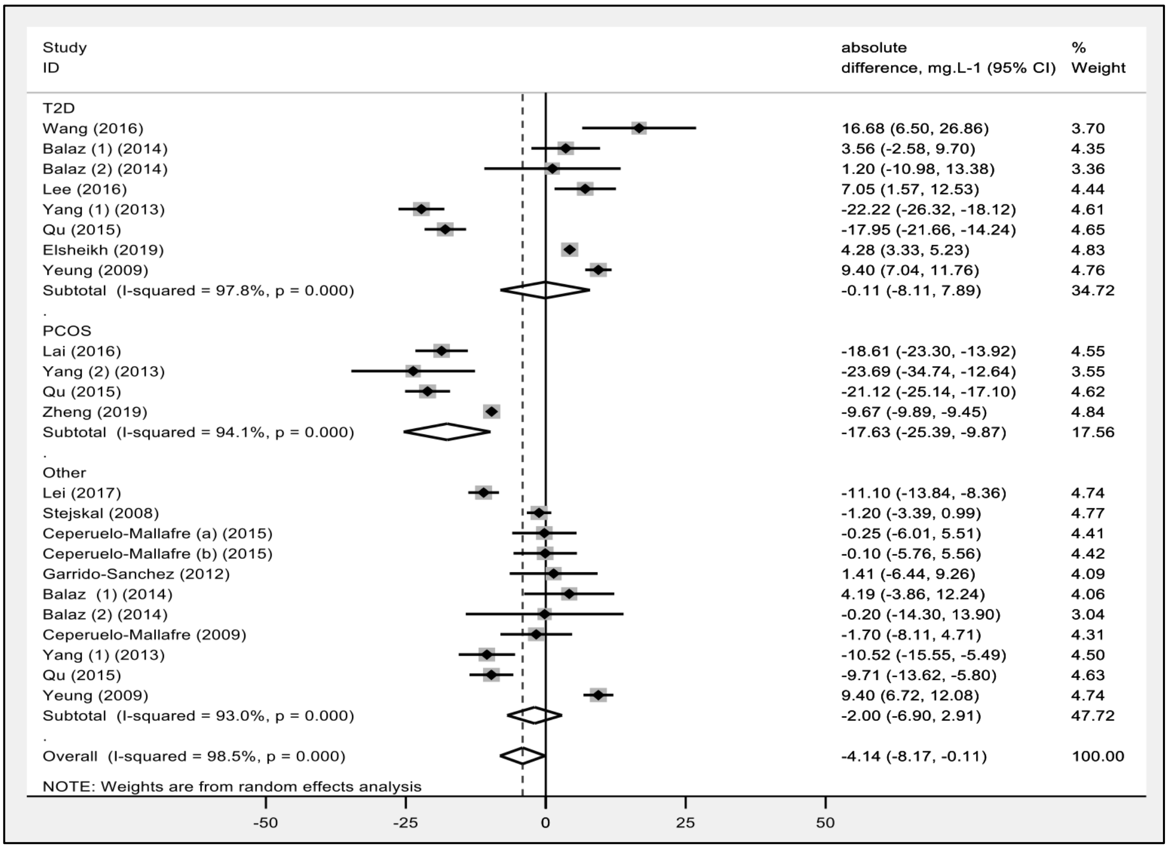

B)

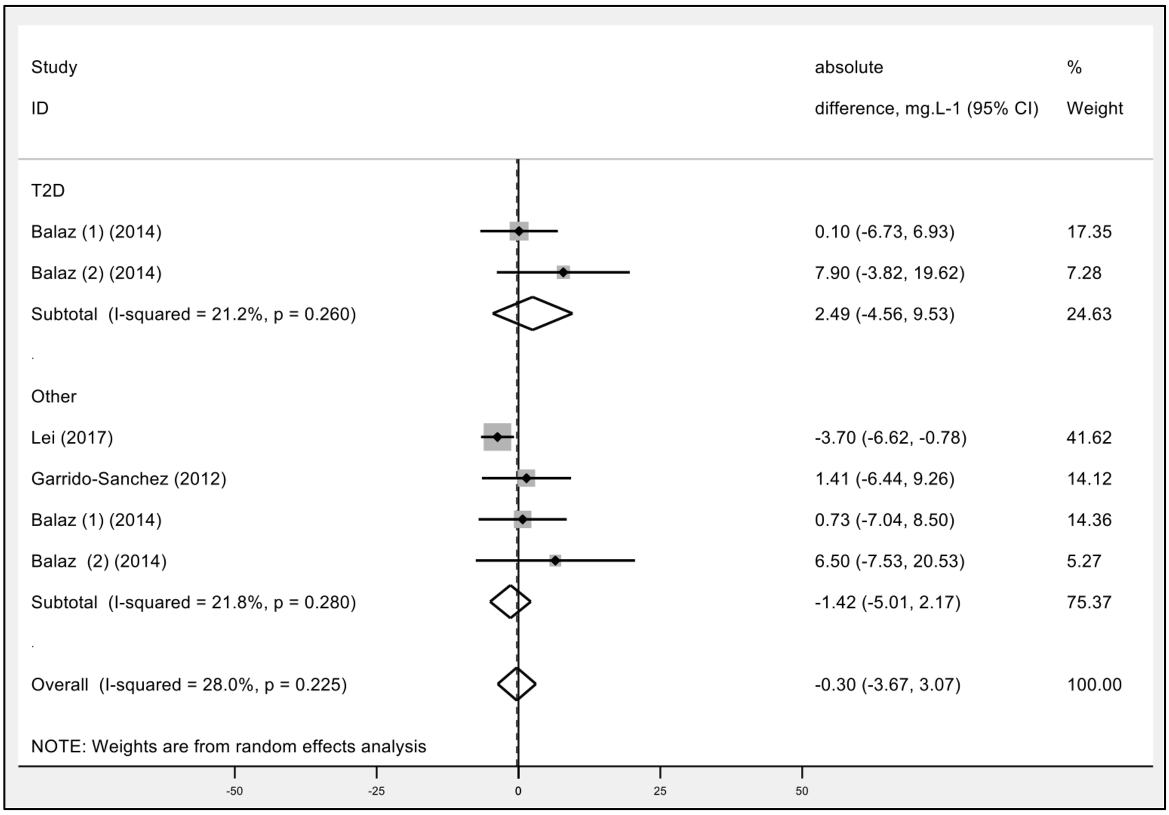

Figure 2. Forest plot from a meta-analysis comparing mean circulating ZAG levels in healthy controls versus cases (T2DM, PCOS or other). Figure A displays absolute pooled data; (1)= cohort one comparison; (2)= cohort two

comparison; (a) = denoted subgroup HOMA-IR 2 and 4 units. (b)= denoted subgroup HOMA-IR >4 units. Figure B displays overweight/ obese data (T2D or other) only; (1)= cohort one comparison; (2)= cohort two comparison.

FG	SN^{N}	1^{489}	$3^{483\,*}$	11^{285}	13^{332}	9^{73}	5^{100}	6^{39}	7^{25}	12^{258}	$14^{66\,\#}$	2^{230}
2h/RG	SN^{N}	1^{489}	$3^{483\,*}$	12^{258}	13^{332}	$14^{66\,\#}$						
FIns	SN^{N}	1^{489}	$3^{483\,*}$	$4^{99\,a}$	9^{73}	11^{285}	13^{332}	$4^{100\,b}$	6^{39}	7^{25}	12^{258}	
HbA1c (%)	SN^{N}	1^{489}	$3^{483\,*}$	$4^{99\,a}$	$4^{100\,b}$	11^{285}	14^{44}					
HOMA-IR	SN^{N}	1^{489}	$3^{483\,*}$	$4^{99\,a}$	9^{73}	11^{285}	13^{332}	$4^{100\,b}$	7^{25}	12^{258}		

Key	
Positive	
Not significant	
Negative	

Figure 3. Summary of the relevant findings from each paper regarding correlational associations between continuous variables and ZAG. SN=study number; N= samples size. 2hG= 2hour glucose; FG= fasting glucose; FIns= fasting insulin; HbA1c= glycated haemoglobin; HOMA-IR= homeostatic model assessment of insulin resistance; RG= random glucose. SN 4 stratified correlations by PCOS[a] (n=99) and control[b] (n=100) subjects and are subsequently reported separately. *SN 3 provided correlation between ZAG index (*Ln*[ZAG/HOMA-IR]) and continuous variables. [#] SN 14 reported correlations only using dysglycaemic individuals which included those with micro-albuminuria and macro-albuminuria. The format used to display this data was adapted from Brocklebank LA, Falconer CL, Page AS, Perry R, Cooper AR. Accelerometer-measured sedentary time and cardio-metabolic biomarkers: a systematic review. Preventive medicine. 2015 Jul 31; 76: 92 102(39).

Figure 4. Funnel Plots

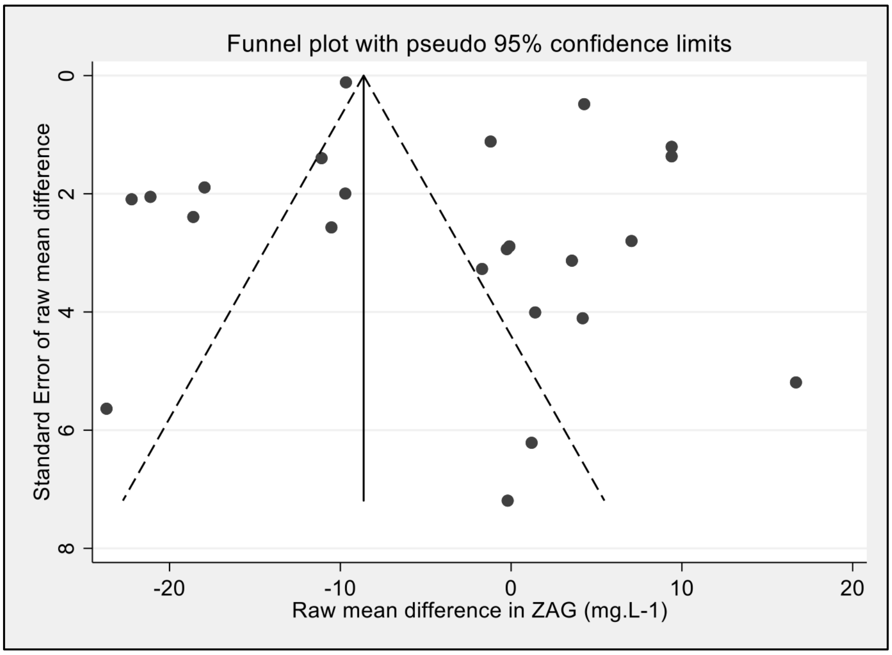

A) Whole cohort. Eggers Test: 3.02 [-0.81, 6.84]; *p*=0.116.

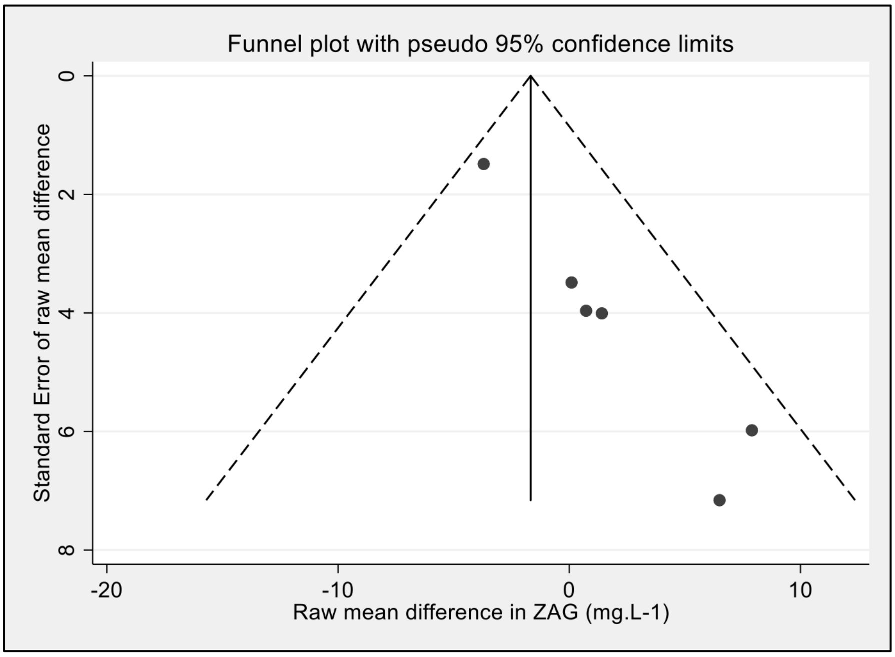

B) Overweight and Obese Participants only. Eggers Test: 2.06 [1.53, 2.59]; *p*<0.001**.**

3. Search methodology PICO model

1. Population of interest

Individuals with type 2 diabetes mellitus (T2DM) but initial search consists of T2DM, type 1 diabetes mellitus (T1DM) and gestational diabetes mellitus (GDM). In addition, the population of interest also includes those with poly-cystic ovary syndrome (PCOS), pre-diabetes, insulin resistance (IR) and metabolic syndrome (MetS).

2. Intervention/ prognosis/ exposure

The main element to be identified in this systematic review is zinc-alpha2-glycoprotein (ZAG) concentration. Therefore, changes in ZAG with an intervention or measurement of ZAG in cross-sectional or longitudinal analysis

3. Comparison

In this systematic review the case group (i.e. T2DM) needed to be compared with a control group (i.e. normal glycaemic tolerance individuals) or be included in a case-series (i.e. with normal glycaemic, obese and pre-diabetic individuals).

4. Outcome

The main outcome assessed was the difference in ZAG between case vs. control or across cases. Also, where necessary, the association between ZAG and different components that are used to assess diabetes risk/ presence.

Search strategy

Published data were obtained by searching of four electronic databases (PubMed, Scopus, Medline and Web of Science) from inception to July 2019. The search was conducted by one reviewer (HMP) and all papers were exported to EndNote X7. The title and abstract were retrieved for each paper and the full text also retrieved for studies considered potentially eligible, each paper identified by the search was independently assessed by two reviewers (HMP and JH) between July and August 2019. Any disagreement or uncertainty regarding the inclusion

of the paper was discussed and consensus was reached. Findings were additionally discussed with a third reviewer (TEY).

When sufficient information was not available for full risk of bias assessment, or if required data were not available in published manuscripts, the authors were contacted and a one-month period was given for a response. If necessary information could not be ascertained and this impacted the inclusion of the paper, it was removed from the final manuscript. The reference lists of included articles were also manually screened for any relevant additional studies.

Inclusion criteria

(1) Written in English or translated into English.

(2) Studies investigating an association between plasma or serum ZAG concentration and markers of glucose metabolism, including IR, T2DM, prediabetes (or associated terms), PCOS or metabolic syndrome.

(3) Case-control/ cohort, cross sectional or prospective observational studies; the only criteria for controls or healthy comparison groups were that they were determined to be metabolically healthy.

(4) Human participants aged ≥18 years.

Key words

List *(a)*:

Zinc-alpha-2-glycoprotein

Zinc-alpha2-glycoprotein

ZA2G

AZGP1

Zn-alpha-2-glycoprotein

ZnGP1

Zinc-α-2-glycoprotein

Zinc- α-2-GP

Zinc adj3 glycoprotein **

Z α2GP

Z α2G

List *(b)*:

T2D

Diabetes Mellitus

Type 2 Diabetes

Type II Diabetes

Adult onset diabetes

NIDDM

Hyperglycaemia

Hyperglycemia

Insulin Resistance

Insulin Sensitivity

Impaired glucose control

Non-diabetic hyperglycaemia

Impaired glucose tolerance

Non-insulin dependent diabetes mellitus

Pre-diabetes

Prediabetes

HOMA-IR

Beta cell dysfunction

B-cell dysfunction

Homeostatic model assessment

Oral Glucose Tolerance Test

OGTT

Diabet*

T2DM

Glycaemic adj4 control**

Glycemic adj4 control **

Glucose

Insulin

HbA1c

A1c

Glycated haemoglobin

*Truncated term

** Subject to change adj for boonlean needed for search in database (i.e. W/, NEAR. ADJn)

Search term strategies

PubMed

((ZAG) OR (zinc-alpha-2-glycoprotein) OR (ZA2G) OR (AZGP1) OR (Zn-alpha-2-glycoprotein) OR (ZnGP1) OR (Zinc-α-2-glycoprotein) OR (Zinc-α-2-GP) OR (Zinc n3 glycoprotein) OR (Zα2G) OR (Zα2GP)) ((T2D) OR (diabetes mellitus) OR (type 2 diabetes) OR (type ii diabetes) OR (adult onset diabetes) OR (niddm) OR (hyperglycaemia) OR (insulin resistance) OR (insulin sensitivity) OR (impaired glucose tolerance) OR (non-insulin dependent diabetes mellitus) OR (pre-diabetes) OR (prediabetes) OR (non-diabetic hyperglycemia) OR (impaired glucose regulation) OR (homa-ir) OR (beta-cell dysfunction) OR (beta cell dysfunction) OR (homeostatic model assessment) OR (oral glucose tolerance test) OR (ogtt) OR (diabet*) OR (T2DM) OR (glycaemic n4 control) OR (glycemic n4 control) OR (insulin) OR (glucose) OR (HbA1c) OR (A1c) OR (glycated haemoglobin))

Scopus

((zag) OR (zinc-alpha-2-glycoprotein) OR (za2g) OR (azgp1) OR (zn-alpha-2-glycoprotein) OR (zngp1) OR (zinc-α-2-glycoprotein) OR (zinc-α-2-gp) OR (zinc W/3 glycoprotein) OR (zα2g) OR (zα2gp)) ((t2d) OR (diabetes AND mellitus) OR (type 2 diabetes) OR (type AND ii AND diabetes) OR (adult AND onset AND diabetes) OR (niddm) OR (hyperglycaemia) OR (insulin AND resistance) OR (insulin AND sensitivity) OR (impaired AND glucose AND tolerance) OR (non-insulin AND dependent AND diabetes AND mellitus) OR (pre-diabetes) OR (prediabetes) OR (non-diabetic AND hyperglycemia) OR (impaired AND glucose AND regulation) OR (homa-ir) OR (beta-cell AND dysfunction) OR (beta AND cell AND dysfunction) OR (homeostatic AND model AND assessment) OR (oral AND glucose AND tolerance AND test) OR (ogtt) OR (diabet*) OR (t2dm) OR (glycaemic W/4 control) OR (glycemic W/4 control) OR (insulin) OR (glucose) OR (hba1c) OR (a1c) OR (glycated AND haemoglobin))

Medline

1.(T2D or diabetes mellitus or type 2 diabetes or type II diabetes or adult onset diabetes or NIDDM or hyperglycemia or hyperglycaemia or insulin resistance or insulin sensitivity or non-insulin dependent diabetes

mellitus or pre-diabetes or prediabetes or non-diabetic hyperglycemia or impaired glucose regulation or HOMA-IR or beta-cell dysfunction or homeostatic model assessment or oral glucose tolerance test or OGTT or diabet* or t2dm or glycaemic NEAR4 control or glycemic NEAR4 control or insulin or glucose or HbA1c or A1c or Glycated haemoglobin or impaired glucose regulation).af. 2. (ZAG or zinc-alpha-2-glycoprotein or ZA2G or AZGP1 or Zn-alpha-2-glycoprotein or ZnGP1 or Zinc NEAR 3 glycoprotein).af. Combine 1 AND 2

Web of Science

1.TS= ((zag) OR (zinc-alpha-2-glycoprotein) OR (za2g) OR (azgp1) OR (zn-alpha-2-glycoprotein) OR (zngp1) OR (zinc-α-2-glycoprotein) OR (zinc-α-2-gp) OR (zinc NEAR3 glycoprotein) OR (zα2g) OR (zα2gp)). 2.TS= ((t2d) OR (diabetes AND mellitus) OR (type 2 diabetes) OR (type AND ii AND diabetes) OR (adult AND onset AND diabetes) OR (niddm) OR (hyperglycaemia) OR (insulin AND resistance) OR (insulin AND sensitivity) OR (impaired AND glucose AND tolerance) OR (non-insulin AND dependent AND diabetes AND mellitus) OR (pre-diabetes) OR (prediabetes) OR (non-diabetic AND hyperglycemia) OR (impaired AND glucose AND regulation) OR (homa-ir) OR (beta-cell AND dysfunction) OR (beta AND cell AND dysfunction) OR (homeostatic AND model AND assessment) OR (oral AND glucose AND tolerance AND test) OR (ogtt) OR (diabet*) OR (t2dm) OR (glycaemic NEAR4 control) OR (glycemic NEAR4 control) OR (insulin) OR (glucose) OR (hba1c) OR (a1c) OR (glycated AND haemoglobin) OR (hyperglycemia)). Combine 1 AND 2.

Some databases required the removal of the alpha (α) and beta (β) symbols and to run the searches.

Numbered strategy example (PubMed)

#1 ZAG

#2 Zinc-alpha-2-glycoprotein

#3 ZA2G

#4 AZGP1

#5 Zn-alpha-2-glycoprotein

#6 ZnGP1

#7 Zinc-α-2-glycoprotein

#8 Zinc-α-2-GP

#9 Zinc n3 glycoprotein

#10 Zα2G

#11 Zα2GP

#12 1 or 2 or 3 or 4 or 5 or 6 or 7 or 8 or 9 or 10 or 11

#13 T2D

#14 diabetes mellitus

#15 type 2 diabetes

#16 type ii diabetes

#17 adult onset diabetes

#18 NIDDM

#19 hyperglycaemia

#20 insulin resistance

#21impaired glucose tolerance

#22non-insulin dependent diabetes mellitus

#23 pre-diabetes

#24 HOMA-IR

#25 beta-cell dysfunction

#26 β cell dysfunction

#27 homeostatic model assessment

#28 oral glucose tolerance test

#29 OGTT

#30 diabet*

#31 T2DM

#32 glycaemic n4 control

#33 glycemic n4 control

#34 hyperglycemia

#35 impaired glucose regulation

#36 non-diabetic hyperglycaemia

#37 insulin

#38 prediabetes

#39 insulin sensitivity

#40 glucose

#41 HbA1c

#42 A1c

#43 glycated haemoglobin

#44 13 or 14 or 15 or 16 or 17 or 18 or 19 or 20 or 21 or 22 or 23 or 24 or 25 or 26 or 27 or 28 or 29 or 30 or 31 or 32 or 33 or 34 or 35 or 36 or 37 or 38 or 39 or 40 or 41 or 42 or 43 or 44

#45 12 and 45

Selection process

The total number of papers imported into Endnote X7 was 1,575.

(1) Duplicate removal

After the removal of duplicates 1,213 manuscripts remained. Any further duplicates identified were later removed during title, abstract/ paper screening.

(2) Screening 1: Title and Abstract

Studies were removed if they met any of the following inclusion criteria:

1) Studies not written in English (English translate accepted)
2) Studies that investigated the trace element zinc (Zn) NOT zinc-alpha2-glycoprotein
3) Studies that did not mention assessing the association between ZAG and a measure of glucose metabolism, insulin resistance/sensitivity or T2DM (from list above or other phrases not included in list) in the title or abstract, as either a primary or secondary outcome
4) reviews/ editorials/ books/ book chapters/ serials
5) Studies not analysing humans

Reference to the association between biomarkers/ proteins and a glycaemic measure in untargeted proteomic studies were included for full screening.

(3) Screening 2: Full paper

Studies that investigated type 1 diabetes, gestational diabetes or diabetes related complications only (including measures of estimated glomerular filtration rate (eGFR)) were removed, as were those that did not measure circulating ZAG (i.e. only reporting urine concentrations or adipose tissue mRNA expression/ protein content).

Data extraction

Data presented in graphical format only

When data was presented in graphical format only, the ZAG concentration values were estimated using commercially available software (Digitizeit, Version 2.3, Bormann I., Braunschweigh, Germany). This software was also used to extract error bar values.

Data presented as median and interquartile range

When data were reported as median (interquartile range), the median value was extracted and used to provide the mean value. The interquartile range (75th quartile/ 25th quartile) was divided by 1.35 to provide an estimated SD (1).

Data presented as standard error of measurement

When data were reported as standard error of measurement, SD was obtained using the following equation (2):

SD= S.E.M * √ n

Converting data to a consistent unit of measurement

ZAG data in this meta-analysis is presented in $mg{\cdot}L^{-1}$. When data were presented in other formats the following conversion were applied:

1 $\mu g{\cdot}mL^{-1}$- = 1 $mg{\cdot}L^{-1}$

1 $ng{\cdot}mL^{-1}$ = 1000 $mg{\cdot}L^{-1}$

Inclusion of sub-sets of participants

In order to remain consistent with the original studies and to provide clear context to our meta-analysis, control groups were used as a comparator even if the study included more than one dysglycaemic group. This applied to the following studies:

Ceperuelo-Mallafré et al. ZAG modulates AKT-dependent insulin signaling in human adipocytes by activation of the PP2A phosphatase (2015). For the purposes of this analysis, both the low (HOMA-IR >2, <4) and high (HOMA-IR >4) HOMA-IR insulin resistant cohorts were compared to insulin sensitive cohort (HOMA-IR <2).

Elsheikh M, Elhefnawy KA, Emad G, Ismail M and Borai M. Zinc alpha 2 glycoprotein as an early biomarker of diabetic nephropathy in patients with type 2 diabetes mellitus (2019). For the purposes of this analysis, we only compared one T2DM cohort (normo-albuminuria) to the control group.

Inclusion of pooled sub-sets of participants

Where appropriate, pooling was performed to ensure results were consistent with those reported in the original studies. This applies to the following studies:

Qu et al. The natural logarithm of ZAG/ HOMA-IR is a better predictor of insulin sensitivity than the product of triglycerides and glucose and other lipid ratios (2015). For the purposes of this analysis, the two control cohorts were pooled and then compared to the dysglycaemic groups.

Wang et al. Adipokine ZAG as a novel urinary biomarker presents earlier than microalbuminuria in diabetic nephropathy (2016). This study only reported a pooled dysglycaemic (T2DM) group that included those with normo-albuminuria, micro-albuminuria and macro-albuminuria.

Overweight/obese restricted meta-analysis inclusion

To be included in this restricted analysis the study had to recruit individuals with overweight or obesity only. Studies were included if they either: specified this in their methodology or the manuscript reported distinct sub-analyses only in individuals with overweight/obesity with or without dysglycaemia. Studies were also included where the mean/median BMI of both case and control groups ≥30 $kg{\cdot}m^2$.

References

1) Higgins JPT, Thomas J, Chandler J, Cumpston M, Li T, Page MJ, Welch VA (editors). Cochrane Handbook for Systematic Reviews of Interventions version 6.0 (updated July 2019). Cochrane, 2019. Available from www.training.cochrane.org/handbook.

2) Higgins JPT, Thomas J, Chandler J, Cumpston M, Li T, Page MJ, Welch VA (editors). Cochrane Handbook for Systematic Reviews of Interventions version 5.1: Part 2: General methods for Cochrane reviews (updated March 2011) Cochrane, 2019. Available from https://handbook-5-1.cochrane.org/chapter_7/7_7_3_2_obtaining_standard_deviations_from_standard_errors_and.htm
